# Construction of chronic inflammation and mitochondrial energy metabolism-associated predictive and therapeutic models for lung adenocarcinoma patients

**DOI:** 10.1007/s12672-026-04822-7

**Published:** 2026-04-05

**Authors:** Yungang Wang, Ling Hao, Lingzi Zhang, Yiru Chen

**Affiliations:** 1 Department of Radiation Oncology Physics & Technology, Cancer Hospital of Shandong First Medical University, Jinan, P.R. China; 2 Department of Neurology Three, The Fifth People’s Hospital of Jinan, Jinan, 250022 Shandong China; 3 Ultrasound Department of No.2 Jinan Maternal and Child Health Hospital, Jinan, 250022 Shandong China; 4 Department of Radiation Oncology, Cancer Hospital of Shandong First Medical University, Jinan, 250022 Shandong China

**Keywords:** Lung adenocarcinoma, Chronic obstructive pulmonary disease (COPD), Mitochondrial energy metabolism, Machine learning, Multi-omics

## Abstract

**Background:**

Lung adenocarcinoma (LUAD) remains the most common subtype of non–small cell lung cancer with poor survival. Dysregulation of mitochondrial energy metabolism (MEM) can be considered as major driver for LUAD pathogenesis among Chronic obstructive pulmonary disease (COPD) patients. Hence, deeper understanding of MEM carcinogenic role for LUAD patients can provide novel insights into LUAD pathogenesis for COPD patients.

**Methods:**

By employing integrative bioinformatic approaches, such as Limma and WGCNA in LUAD bulk profile (GSE32863), we first identified COPD and MEM (CM)-associated DEGs for LUAD patients, and these DEGs can divide LUAD patients from TCGA-LUAD cohort into 2 risk groups. Next, Lasso-cox regression and multi-variate cox regression identified CM-associated predictive model and hub gene for LUAD patients both in TCGA-LUAD cohort and GSE13213. Indeed, hub gene molecular and immune characters were estimated at LUAD bulk level (TCGA-LUAD cohort) and single-cell level (GSE203360). Besides, drug sensitivity and molecular docking analysis confirmed potential therapeutic agent targeting hub gene for the treatment of LUAD.

**Results:**

8 CM-associated gene signatures can divide LUAD patients into 2 molecular subgroups and guide the prognostic model construction for LUAD patients. TPI1 can be considered as CM-associated hub gene involved in LUAD progression. 17-AAG can be considered as drug reproposing framework for the treatment of LUAD.

**Conclusion:**

This study first highlighted the predictive and therapeutic potentials of CM, and highlighted TPI1 pathogenic role for LUAD patients.

## Introduction

 Lung adenocarcinoma (LUAD), the most common subtype of non-small cell lung cancer (NSCLC), accounts for 40–45% of cases worldwide [1]. Despite therapeutic advances, the 5-year survival remains < 20% overall and < 10% in advanced disease, highlighting the need for robust biomarkers to refine prognosis and guide therapy [2].

Chronic obstructive pulmonary disease (COPD) is an independent risk factor for LUAD, increasing lung cancer risk even among never-smokers [3]. COPD pathology, such as chronic inflammation, oxidative stress, and airway remodeling contribute to the immune imbalance of lung, leading to the carcinogenesis of LUAD [4]. Recent molecular studies show overlapping pathways between COPD and LUAD, including extracellular matrix remodeling and immune signaling. A unifying mechanism is mitochondrial energy metabolism (MEM) [5]. In COPD, epithelial and immune cells display mitochondrial dysfunction with impaired oxidative phosphorylation (OXPHOS), excess mitochondrial reactive oxygen species (mtROS), and defective mitophagy, perpetuating chronic inflammation and malignant transformation [6]. Besides, the COPD and MEM-associated SLC7A11 and GYS1 also contribute to the pathogenesis and tumor microenviroment (TME) heterogeneity of LUAD patients [7]. Besides, as another COPD and MEM regulator, epidermal growth factor receptor (EGFR) mutation can be considered as a key oncogenic driver in regulation of immunosuppressive TME for LUAD patients [8, 9]. In LUAD, metabolic reprogramming enables dynamic switching between glycolysis and OXPHOS, sustaining growth, remodeling the immune microenvironment, and influencing therapeutic response. Significantly, COPD and MEM-associated SLC25A11 can modulate metabolic reprogramming of LUAD and immunotherapy efficacy for LUAD patients [10]. Together, MEM can be considered as a linker between COPD and LUAD tumor biology.

**Fig. 1:**
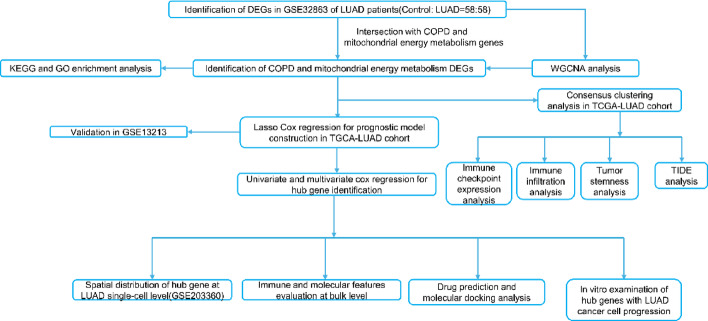
The flowchart of this study

In this study, by integration of integrative bioinformatic pipelines and multi-omics, we first identified COPD and MEM(CM)-associated DEGs and molecular subgroups for LUAD patients. Besides, we also highlighted that TPI1 can be considered as CM-associated prognostic indicator involved in pathogenesis of LUAD. 17-AAG, a small compound, also can be considered as therapeutic reproposing strategy targeting TPI1 for the treatment of LUAD. In conclusion, our study first revealed the CM-associated mechanisms and corresponding prognostic and therapeutic potentials for LUAD patients. We illustrated the workflow of this study in Fig. 1.

## Materials and methods

### Source of data

The gene expression profile GSE32863 (based on GPL6884, including 58 LUAD lung tissues and 58 paired para-cancer tissues) and GSE13213 (based on GPL6480, including 117 LUAD samples) were downloaded from the Gene Expression Omnibus (GEO) database via GEOquery package of R [11, 12]. These 2 dataset was normalized and standardized by Limma package of R, of which GSE32863 normalized results was visualized by PCA plot generated by ggplot2 package of R [13, 14]. Additionally, we downloaded TCGA-LUAD expression profile and corresponding clinical information from TCGA database [15]. After normalized and standardized by edgeR package of R, we finally acquired 598 LUAD lung tissues and 59 normal lung samples for further analysis [16]. GSE13213 In addition, COPD gene list was acquired from CTD and Genecard database respectively with threshold > 1 (Genecard database acquisition), and MEM-related gene list were obtained from Genecard database with threshold > 1 [17, 18].

### Identification of DEGs

Differentially expressed genes (DEGs) in GSE32863 were identified via Limma package of R with the following criteria: p value < 0.05 and |log fold change (FC)| > 0.5 [19]. DEGs in GSE32863 were visualized by volcano map and heatmap via ggplot2 and complexheatmap package of R [14, 20]. Subsequently, DEGs in GSE32863 were intersected with COPD gene list and MEM gene list to acquire CM-associated DEGs. To further investigate the potential biological roles of the CM-associated DEGs, we performed KEGG and GO enrichment analysis in accordance with KEGG and GO gene set downloaded from MSIGDB database via ClusterProfiler package of R [21].

### WGCNA analysis

In GSE32863, we utilized the “WGCNA” package in R to construct a gene co-expression network, aiming to investigate the associations between genes and LUAD characteristics [22, 23]. First, we filtered out the 50% of genes with the lowest median absolute deviation (MAD) [22, 23]. Next, Pearson’s correlation matrices were calculated for all gene pairs, and a weighted adjacency matrix was generated using the average linkage method combined with a weighted correlation coefficient [22, 23]. The “soft” thresholding parameter (β) was applied to obtain the adjacency, which was then converted into a topological overlap matrix (TOM) [22, 23]. To group genes with similar expression profiles into distinct modules, average linkage hierarchical clustering was performed based on the TOM-derived dissimilarity, with a minimum module size set at 60 genes [22, 23]. Finally, we evaluated the dissimilarity among module eigengenes, determined an appropriate cut line for the module dendrogram, and merged several modules [22, 23]. The module illustrated highest correlation with the LUAD was selected and then extracted molecules in this module. Next, we intersected CM-associated DEGs with genes in this module for identification of CM-associated shared DEGs.

### Lasso and Cox regression model construction

In TCGA-LUAD cohort, we utilized Least Absolute Shrinkage and Selection Operator (Lasso)-Cox regression technique for identifying CM-associated prognostic model with screening criteria as deviance and 10-fold cross validation via glmnet package of R [24]. Besides, Overall survival(OS) Lasso-cox regression model performance were illustrated by Time-dependent ROC plot and KM plot via survival and rms package of R [25]. Next, hub gene authentication was assessed by univariate and multivariate cox regression analysis with LUAD patient clinical information via survival package of R [25]. Besides, hub gene prognostic and expression values with clinical information of LUAD were assessed by nomogram and calibration analysis via survival and rms package of R [26]. The dignostic value of TPI1 for LUAD patients was assessed by ROC analysis conducted by pROC package of R [23]. After identifying hub gene, CIBERSORT package of R was employed in TCGA-cohort for investigation of the association between hub gene and immune proportion in LUAD patients [27]. Additionally, the co-expression of immune checkpoints and hub gene was also estimated in TCGA-LUAD cohort. Next, ssGSEA algorithm was utilized for identification of correlation between tumor phenotypes and hub gene expression. The promoter methylation of hub gene in LUAD patients was assessed in UALCAN database [28]. Besides, Tumor Mutational Burden (TMB) of hub gene in LUAD patients was also assessed in TCGA-LUAD cohort [29]. Next, transcriptional factors (TFs) targeting hub gene was enriched via several database, such as KnockTF, GTRD, CHIP_Atlas and ENCODE, and co-expression patterns of these TFs with TPI1 were analyzed in TCGA-LUAD cohort [30–33]. Besides, single-gene GSEA enrichment analysis was enriched in TCGA-LUAD cohort for estimation of hub gene molecular and biological functions for LUAD patients via the ClusterProfiler package of R in accordance with hallmark gene set downloaded from MSIGDB database [21].

### Consensus clustering

To identify molecular subgroups associated with the CM for LUAD patients, we conducted consensus clustering analysis in TCGA-LUAD cohort based on CM-associated DEGs via ConsensusClusterPlus package in the R software [34]. In each iteration, 80% of the samples were randomly chosen, and clustering was performed based on Pearson correlation distance. This process was repeated 10 times, with results aggregated across iterations to ensure a robust and stable classification [34]. Furthermore, tumor stemness was evaluated using the OCLR algorithm implemented in R [35]. The OS clinical outcomes between subgroups were analyzed via KM analysis of R [25]. To explore the potential implications for precision medicine within CM-related subgroups, we analyzed and visualized immune infiltration and immune checkpoint expression between CM-associated subgroups via CIBERSORT package of R [27]. Additionally, clinical parameters were compared between the subgroups. TIDE analysis was performed for analyzing the immunotherapy difference between CM-associated subgroups [36]. Besides, GSEA enrichment analysis was performed for identification of molecular and biological difference between subgroups via clusterProfiler package of R in accordance with the Hallmark gene set downloaded from MSIGDB database [21].

### Single-cell analysis

We downloaded the LUAD single-cell data (GSE203360, based on GPL20705, including 6 LUAD tissue samples) from GEO database [37]. Subsequently, we utilized the Seurat package in R to perform quality control (QC) evaluations [38]. The established quality thresholds included filtering out genes detected in fewer than 3 cells, discarding cells with a total of less than 50 identified genes, and removing cells where mitochondrial gene expression accounted for 5% or more of the total expression [38]. Standard preprocessing and normalization procedures, such as percentage feature set filtering, SCTransform, principal component analysis (PCA), neighbor detection, clustering, uniform manifold approximation and projection (UMAP) for dimensionality reduction, and marker gene identification, were executed using the Seurat package in R [38]. Following this, cell annotation was carried out based on established cell markers obtained from the Cellmarker 2.0 database [39].

### Drug prediction and molecular docking

GSCA database was employed to analyze the drugs targeting hub gene and conducted drug sensitivity analysis [40]. After confirming optimal drug, Oncopredict package of R was performed for identification of agent sensitivity to LUAD [41]. Molecular docking was employed to assess the interactions between drugs and proteins. The three-dimensional structures of the target proteins were retrieved from the RCSB Protein Data Bank (PDB IDs: 7UXV for TPI1), while the ligand structures were sourced from the Pubchem database (Compound CID: 6505803 for 17-AAG) [42, 43]. Subsequently, molecular docking simulations were carried out to estimate the binding affinity between the target proteins and the compounds [44]. The procedure involved initially using PyMOL software (Version 2.6.0) to eliminate water molecules and existing ligands, preserving only the protein backbone [44]. Following this, AutoDock Vina (Version 4.2.6) was utilized to identify potential binding sites on the protein surface and conduct flexible docking. The software computed docking scores and binding affinities (expressed as Vina scores in kcal/mol) for each identified binding cavity [44]. The optimal cavity was ranked according to their binding energy, and the conformation with the lowest binding energy was selected for further visualization in PyMOL [44]. This visualization highlighted the positions of hydrogen bonds formed during ligand binding [44].

### Cell lines and culture

The A549, BEAS-2B, and HEK 293T cell lines were obtained from the Shanghai Academy of Biological Sciences, situated in Shanghai, China. The A549 and BEAS-2B cell lines were grown in a complete Roswell Park Memorial Institute (RPMI) 1640 medium, which was supplemented with 1% of a penicillin-streptomycin antibiotic mixture and 10% fetal bovine serum (FBS, Gibco). Conversely, the HEK 293T cell line was sustained in Dulbecco’s Modified Eagle Medium (DMEM), which was also supplemented with a 1% penicillin-streptomycin mixture and 10% FBS. All procedures for cell passage were performed in a controlled environment at 37 °C with 5% CO2 in a humidified incubator.

### RNA extraction and q-RT-PCR

Total RNA extraction was conducted using TRIzol reagent (TaKaRa, Beijing, China), followed by the assessment of its concentration, purity, and integrity through a NanoDrop spectrophotometer (Thermo Scientific, Waltham, MA, USA). For the reverse transcription process, 1 µg of total RNA was employed in conjunction with HiScript II Q RT SuperMix for qPCR (+ gDNA wiper) and a gDNA eraser (Vazyme, Shanghai, China). The concentration, purity, and integrity of the resultant complementary DNA (cDNA) were subsequently evaluated with the same NanoDrop spectrophotometer. Quantitative reverse transcription polymerase chain reaction (qRT-PCR) was carried out utilizing SYBR Green MasterMix (11203ES50, YEASEN, Shanghai, China) and StepOne Software v.2.3 (Applied Biosystems, Carlsbad, CA, USA) over 40 cycles, including three biological replicates for each specimen. The data analysis was performed using the ∆∆Ct (cycle threshold) method, normalizing against the expression levels of the housekeeping gene, GAPDH. The primer sequences employed in the qRT-PCR procedures are as follows:

TPI1:

F: 5′- CCCAGGAAGTACACGAGAAG-3′.

R: 5′-CAGTCACAGAGCCTCCATAAA-3′.

GAPDH.

F 5′-GAGAAGGCTGGGGCTCATTT-3′.

R 5′-ATGACGAACATGGGGGCATC-3′.

### Silencing via shRNA and overexpression

The target sequences for silencing TPI1 were as follows:

Sh1:

5′ TGATGTGGATGGCTTCCTTGT- 3′.

All sequences were inserted into the pLKO.1 lentiviral vector. Specifically, the plasmids encoding shRNA were co-transfected with the VSV-G envelope plasmid and the psPAX packaging plasmid into HEK293T cells. This procedure was performed using Lipofectamine 2000 (Thermo Fisher Scientific) following the manufacturer’s instructions. The culture medium was replaced the following day, and the lentivirus-containing supernatants were harvested three days after transfection, filtered, and then applied to infect target cells in the presence of 4 µg/ml polybrene (Sigma-Aldrich). Subsequently, A549 cells were seeded at a density of 5 × 10^4 cells per well in 24-well plates and cultured until they reached 50–70% confluence. The standard culture medium was then replaced with a diluted sh-TPI1 lentiviral stock solution for transfection. After a 72-hour incubation, the cells were trypsinized and washed with phosphate-buffered saline (PBS). The cells were then plated onto 10 cm culture dishes at a density of 500 cells per dish, and selection was performed with puromycin (Thermo Scientific) for three weeks. Surviving clones were identified by the formation of cloning rings, after which they were expanded and subcloned using the limiting dilution method.

### Cell proliferation assays

Cells that were in the logarithmic growth phase underwent trypsinization, followed by counting and subsequent distribution into 96-well plates at a density of 3000 cells per well (*n* = 6). After incubating the plates for specific time intervals (4, 24, 48, 72, 96, and 120 h), 10 µL of CCK-8 reagent was added to each well, and the plates were further incubated for an additional 2 h. Absorbance measurements were recorded at 450 nm using a microplate reader. The rate of cell proliferation was calculated using the equation (A_d − A_blank_d) / (A_4h − A_blank_4h). All experiments were performed in triplicate to enhance the reliability of the findings.

### Transwell assays

The study utilized Transwell chambers manufactured by Corning Inc., located in New York, USA. Each chamber was filled with 150 µl of a cell suspension containing a total of 10,000 cells. In the lower chamber, 600 µl of culture medium supplemented with 10% fetal bovine serum was introduced. After a 24-hour incubation period, the non-migratory cells were meticulously removed, and the cells that had migrated were stained using a 0.25% crystal violet solution for a duration of 10 min. Subsequently, the chambers were rinsed with phosphate-buffered saline (PBS) and images were captured for analysis. For the in vitro cell invasion assay, Transwell chambers pre-coated with a matrix gel at a concentration of 100 micrograms per square centimeter, obtained from Thermo Fisher Scientific, were employed.

### Western blotting (WB)

Following the administration of various treatment modalities, the cells underwent rinsing with ice-cold phosphate-buffered saline (PBS) obtained from Hyclone, Seattle, WA, USA, and were subsequently collected through careful scraping techniques. The extraction of total protein was accomplished by lysing the cells utilizing radioimmunoprecipitation assay (RIPA) lysis buffer from Beyotime, Shanghai, China, which was supplemented with a combination of phosphatase inhibitors and protease inhibitors, also sourced from Beyotime, China. The resultant cell lysates underwent centrifugation at 14,000× g for a duration of 15 min at a temperature of 4 ◦C. Following centrifugation, the lysates were denatured for 10 min in a 5× SDS-PAGE loading buffer provided by Beyotime, China. Subsequently, the proteins were separated through SDS-PAGE and transferred onto polyvinylidene fluoride (PVDF) membranes, also from Beyotime, China, for the analysis via Western blotting. The membranes were subjected to blocking with NcmBlot blocking buffer (NCM Biotech, Suzhou, China) for 10 min. They were then incubated with primary antibodies for a period of 8 h at 4 ◦C, after which they were diluted in a 5% bovine serum albumin (BSA) solution, purchased from Solarbio, Beijing, China. Following this step, the membranes were treated with secondary antibodies (ThermoFisher, Waltham, MA, USA) that were diluted in WB secondary antibody diluent solution from Beyotime, Shanghai, China, at a dilution ratio of 1:1000 for 2 h at room temperature. Protein detection was performed utilizing an enhanced chemiluminescence (ECL) substrate from Thermo Fisher, Waltham, MA, USA. The quantification of protein expression was executed by analyzing the band densities of the target proteins through ImageJ software version 1.57, with the analysis being based on the density values in relation to the GAPDH protein. The primary antibodies utilized in this investigation were as follows: TPI1 (ab96696, ABCAM, USA: 1:2000), and GAPDH (ab181602, Abcam, USA,1:1000).

### Statistical analysis

In the bioinformatics analysis, all statistical assessments were performed using R software (version 4.2.2). Differences in the proportions of immune-infiltrating cells within the TME were evaluated using the Wilcoxon test. Relationships among various variables were examined through Pearson correlation analysis. Statistical significance was defined as a p-value or false discovery rate (FDR) below 0.05. Data are reported as mean ± standard deviation (SD), with significance levels indicated as *p < 0.05, **p < 0.01, and ***p < 0.001. For the experimental section, all statistical analyses were carried out using GraphPad Prism (version 8.0.2). Each experiment was conducted with at least three biological replicates, and results are expressed as mean ± SD. Differences between two datasets were assessed using either two-way analysis of variance (ANOVA) or Student’s t-test, with statistical significance set at *p* < 0.05.

## Results

### Identification of CM-related DEGs for LUAD patients

**Fig. 2:**
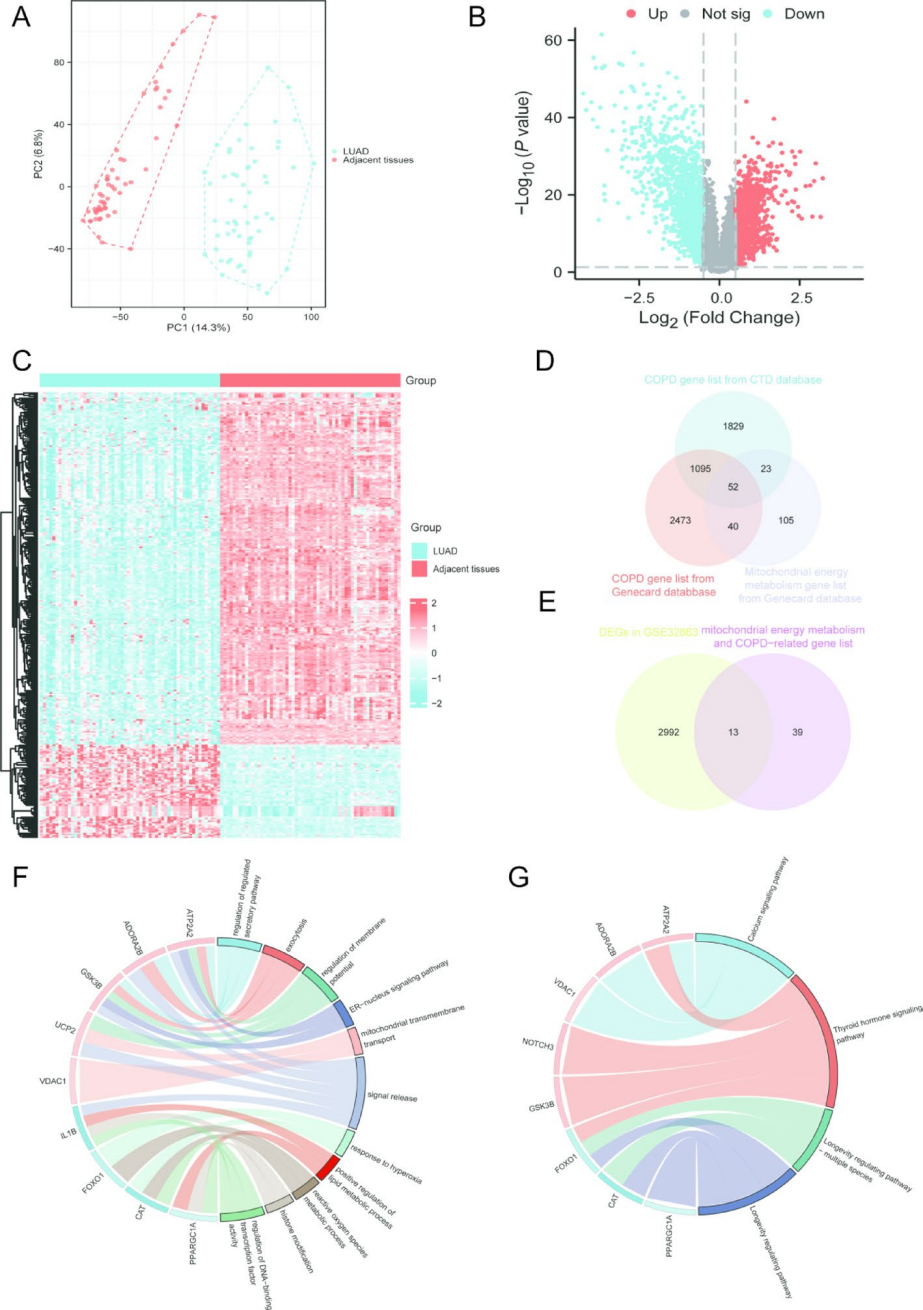
Identification of CM-associated DEGs. **A** PCA plot of GSE32863. **B** Volcano plot of DEGs in GSE32863. **C** Heatmap of DEGs in GSE32863. **D** CM-associated gene list identification. **E** CM-associated DEGs identification in GSE32863. **F–G** GO and KEGG enrichment analysis of CM-associated DEGs in GSE32863

In GSE32863, PCA plot illustrated clear separate between adjacent normal tissues and LUAD samples after normalization (Fig. 2A). Next, 1470 up-regulated DEGs and 1535 down-regulated DEGs were identified in GSE32863 (Fig. 2B–C). Subsequently, we integrated COPD-related genes from the CTD and Genecard database and then intersected with MEM-associated gene list from Genecard database to identification of 52 CM-associated DEGs for LUAD patients (Fig. 2D). Next, CM-associated gene list was intersected with DEGs in GSE32863 for identification of 13 CM-associated DEGs (Fig. 2E). GO and KEGG enrichment pathway analyses were performed for identification of CM-associated DEGs biological significance, and results demonstrated significant enrichment in 16 functions and signaling pathways closely associated with tumorigenesis and metabolic regulation (Fig. 2F-G).

### WGCNA analysis for COPD and MEM module recognition in LUAD patients

**Fig. 3:**
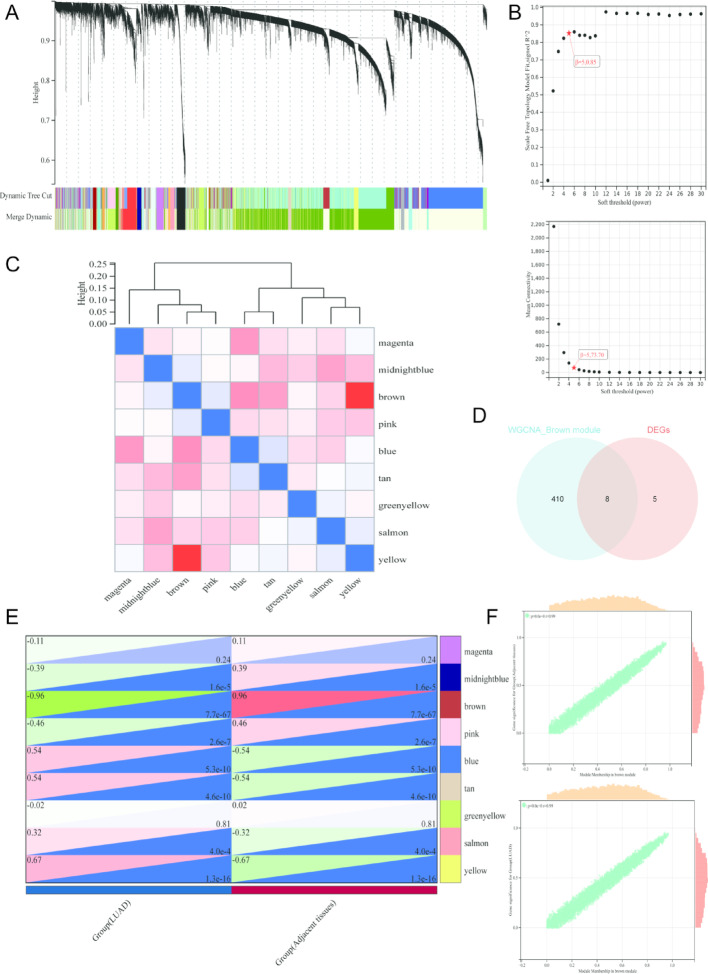
WGCNA analysis for identification of co-expression CM-associated DEGs in LUAD patients. **A** Gene co-expression modules with different colors under the gene tree. **B** β soft threshold chosen based on the scale independence and average connectivity. **C** Heatmap of eigengene adjacency. **D** Venn diagram of module genes and DEGs. **E** Correlation analysis between gene co-expression modules and LUAD or adjacent tissues. **F** Correlation analysis between gene significance and module membership in the brown module

We constructed co-expression modules for LUAD patients based on GSE32863 via WGCNA analysis. First, by analyzing the scale-free topology and mean connectivity, we determined the soft-thresholding power parameter (β = 5,0.85 and 5,73.70) as the optimal threshold to ensure that the network conformed to scale-free characteristics (Fig. 3B). On this basis, hierarchical clustering was applied to the gene expression profiles, which identified 9 co-expression modules with similar expression patterns, each represented by a distinct color (Fig. 3A and C). To further explore the modules most closely associated with LUAD, we performed module–trait correlation analysis and found that the brown module was significantly positively correlated with both LUAD and adjacent normal tissues (*R* = 0.96, *p* < 0.001 for LUAD samples and *R*= -0.96, *p* < 0.001 for normal samples). By intersecting the genes from the brown module with the CM-associated DEGs, we identified 8 overlapping genes (CM-associated shared DEGs) for LUAD patients (Fig. 3E-F).

### CM-related prognostic model and indicators identification for LUAD patients

**Fig. 4:**
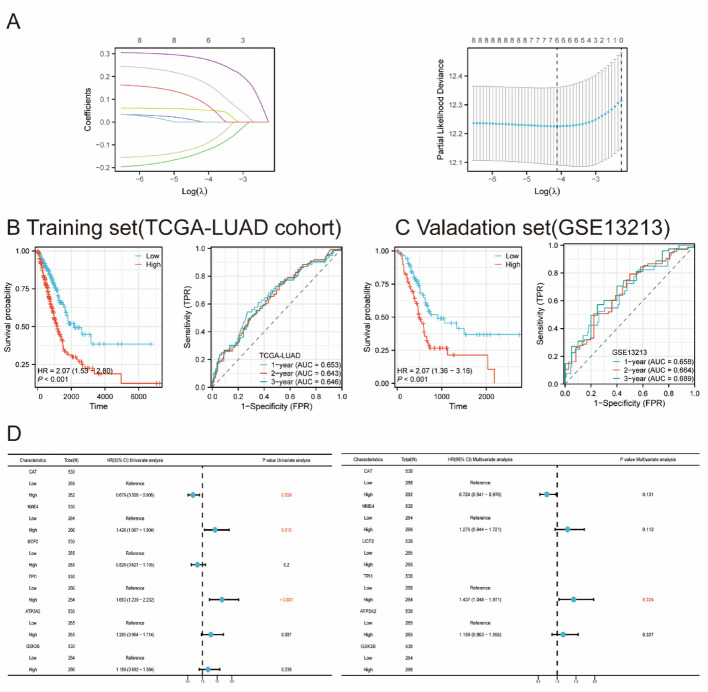
Construction of CM-associated prognostic model and hub genes for LUAD patients. **A** Lasso regression results. **B** Prognostic model construction in TCGA-LUAD cohort. **C** Prognostic model construction in GSE13213 cohort. **D** Univariate and multivariate Cox regression analysis of six prognostic genes in LUAD patients

As shown in Fig. 4A, we applied LASSO-Cox regression analysis to construct CM-associated prognostic model. By selecting the optimal λ value through cross-validation, we identified satisfied CM-related prognostic model and risk stratification for LUAD patients in TCGA-LUAD cohorts, and validated in GSE13213 (Fig. 4B-C). After Univariate multivariate Cox regression adjustment, only TPI1 remained an independent prognostic factor (HR = 1.437, 95% CI: 1.048–1.971, *p* = 0.024, Fig. 4E). Moreover, comprehensive analysis incorporating clinical features confirmed that high TPI1 expression retained significant prognostic value after adjustment (HR = 1.254, *p* = 0.038, Fig. 4F).

### Prognostic value evaluation of TPI1 in in LUAD patients

**Fig. 5:**
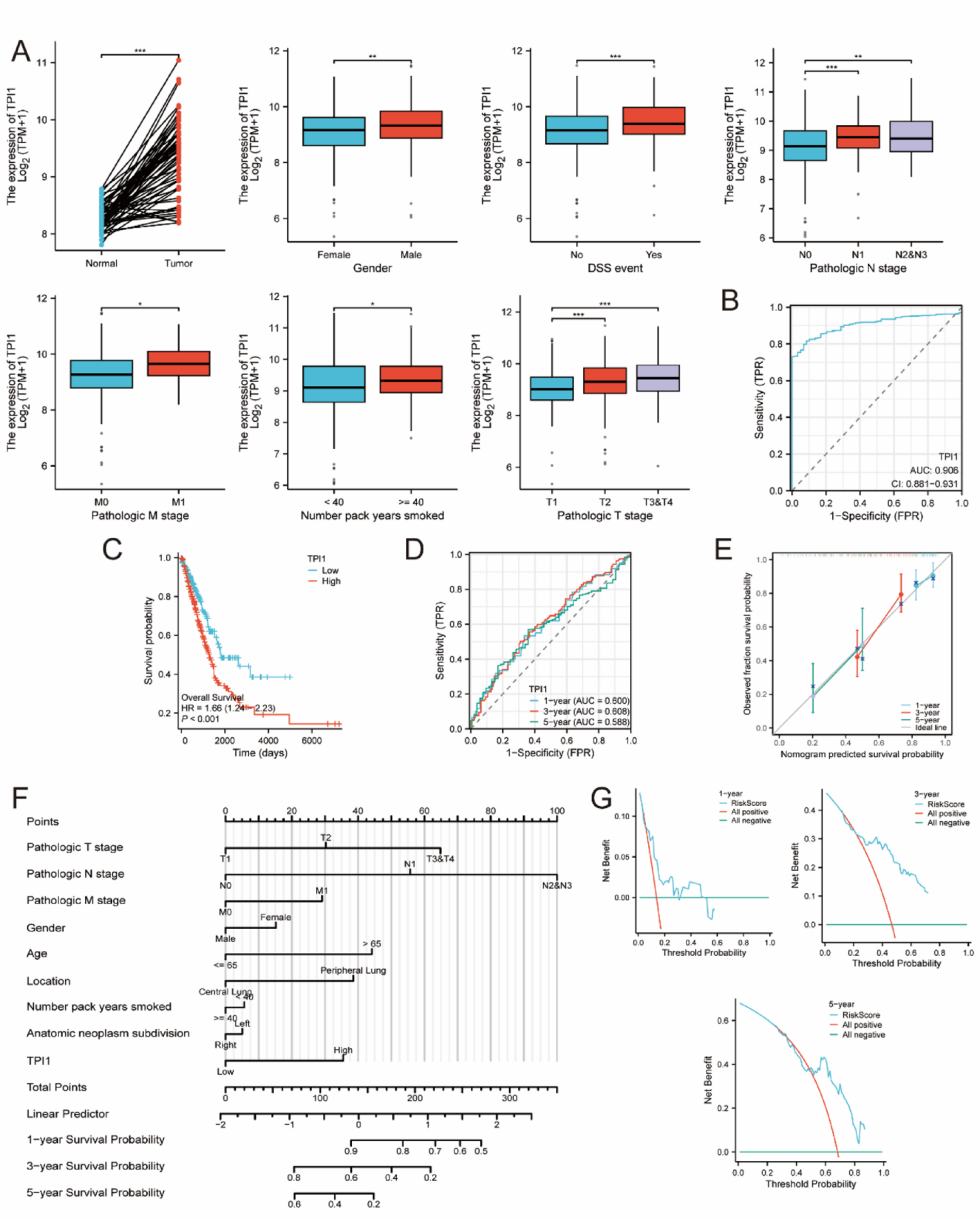
Molecular and prognostic forecasting role of TPI1 in LUAD patients. **A** The expression of TPI1 in normal samples compared to tumor samples; The expression of TPI1 in different stages in LUAD samples; The expression of TPI1 in Overall Survival event in LUAD samples; The expression of TPI1 in Disease-Specific Survival event in LUAD samples; The expression of TPI1 in different gender LUAD patients; The expression of TPI1 in different smoking exposure LUAD patients. **B** Evaluation of TPI1 expression with LUAD onset. **C**,** D** Prognostic evaluation of TPI1 expression in LUAD patients. **E**,** F** Nomogram and calibration plots of TPI1 in LUAD. **G** DCA plot of TPI1

As shown in Fig. 5A, the expression of TPI1 was significantly higher in LUAD tissues compared with normal tissues. Elevated TPI1 expression was strongly associated with pathological stage progression (T, N, and M stages), poor prognosis (including both OS and Disease-Specific Survival [DSS]), male patients, and heavy smoking history. High TPI1 expression was also associated with LUAD onset (Fig. 5B). To systematically evaluate the prognostic value of TPI1, we conducted KM survival analysis, time-dependent ROC, nomogram prediction, and decision curve analysis (DCA). The KM curve showed that patients with high TPI1 expression had significantly shorter OS time compared with those with low expression (Fig. 5C-D). The nomogram model with calibration suggested that TPI1 had strong predictive power for individualized survival prediction, while the DCA analysis further validated the clinical utility of the TPI1-based predictive model (Fig. 5E-G).

### Immune and molecular features of TPI1 in LUAD patients

**Fig. 6:**
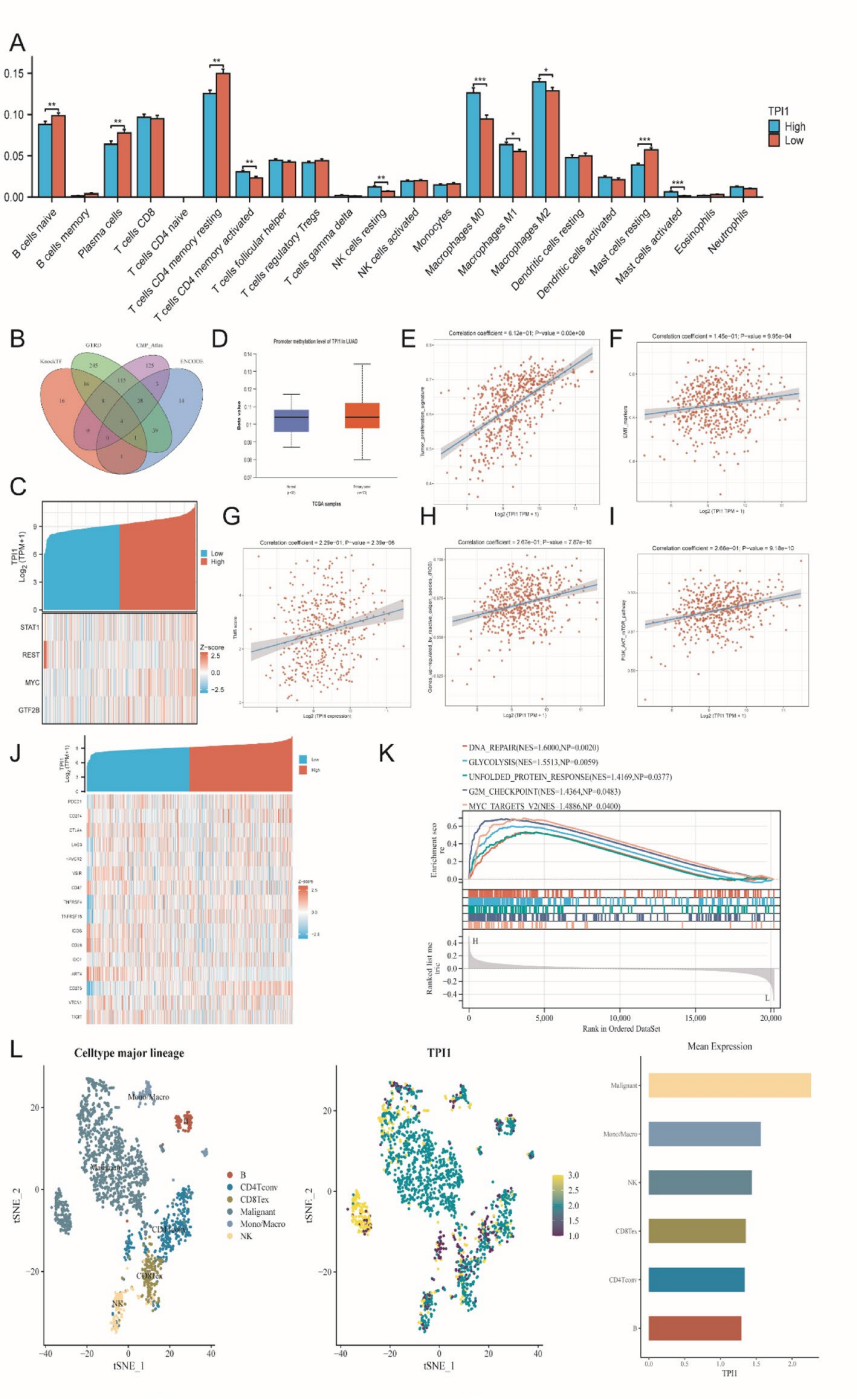
Immune and single-cell features of TPI1. **A** Immune infiltration Immune infiltration Boxplot of TPI1 in LUAD. **B**,** C** TF identification for TPI1 in TCGA-LUAD cohort. **D** DNA methylation analysis of TPI1 in LUAD. **E–I** TPI1 expression and tumor features and TMB in LUAD. **J** Immune checkpoint co-expression heatmap of TPI1 in LUAD. **K** Single-gene GSEA analysis of TPI1 in LUAD. **L** Single-cell analysis of TPI1 in LUAD

Using the CIBERSORT algorithm, we found that TPI1 expression was closely associated with multiple immune cell infiltrations, such as B cells, plasma cells, CD8 + T and CD4 + T cells, and several myeloid cells, indicating that TPI1 differentially expressed can lead to a immunosuppressive microenviroment for LUAD patients (Fig. 5A). Next, by integrating the KnockTF, GTRD, ChIP_Atlas, and ENCODE databases, we identified STAT1, REST, MYC, and GTF2B as potential upstream transcription factors of TPI1, suggesting that its overexpression may be driven by coordinated transcriptional regulation (Fig. 6B-C). Further analysis revealed that multiple immune checkpoint genes were significantly upregulated in the TPI1 high-expression group (Fig. 6J). In addition, TPI1 expression in LUAD tissues was associated with increased promoter methylation levels (Fig. 6D), and showed strong correlations with higher tumor mutation burden, enhanced proliferative activity, PI3K-AKT signal and upregulation of oxidative stress-related genes (Fig. 6E-I). At the molecular level, single-cell transcriptomic analysis and GSEA analysis demonstrated that TPI1 was highly expressed in malignant tumor cells and significantly enriched in critical pathways, including DNA repair, glycolysis, unfolded protein response, G2M checkpoint, and MYC targets (Fig. 6K and L). These findings indicate that TPI1 may play a central role in both metabolic reprogramming and immune microenvironment regulation, thereby promoting LUAD progression by enhancing metabolic activity and proliferative capacity.

### Therapeutic agent identification targeted TPI1 in LUAD patients

**Fig. 7:**
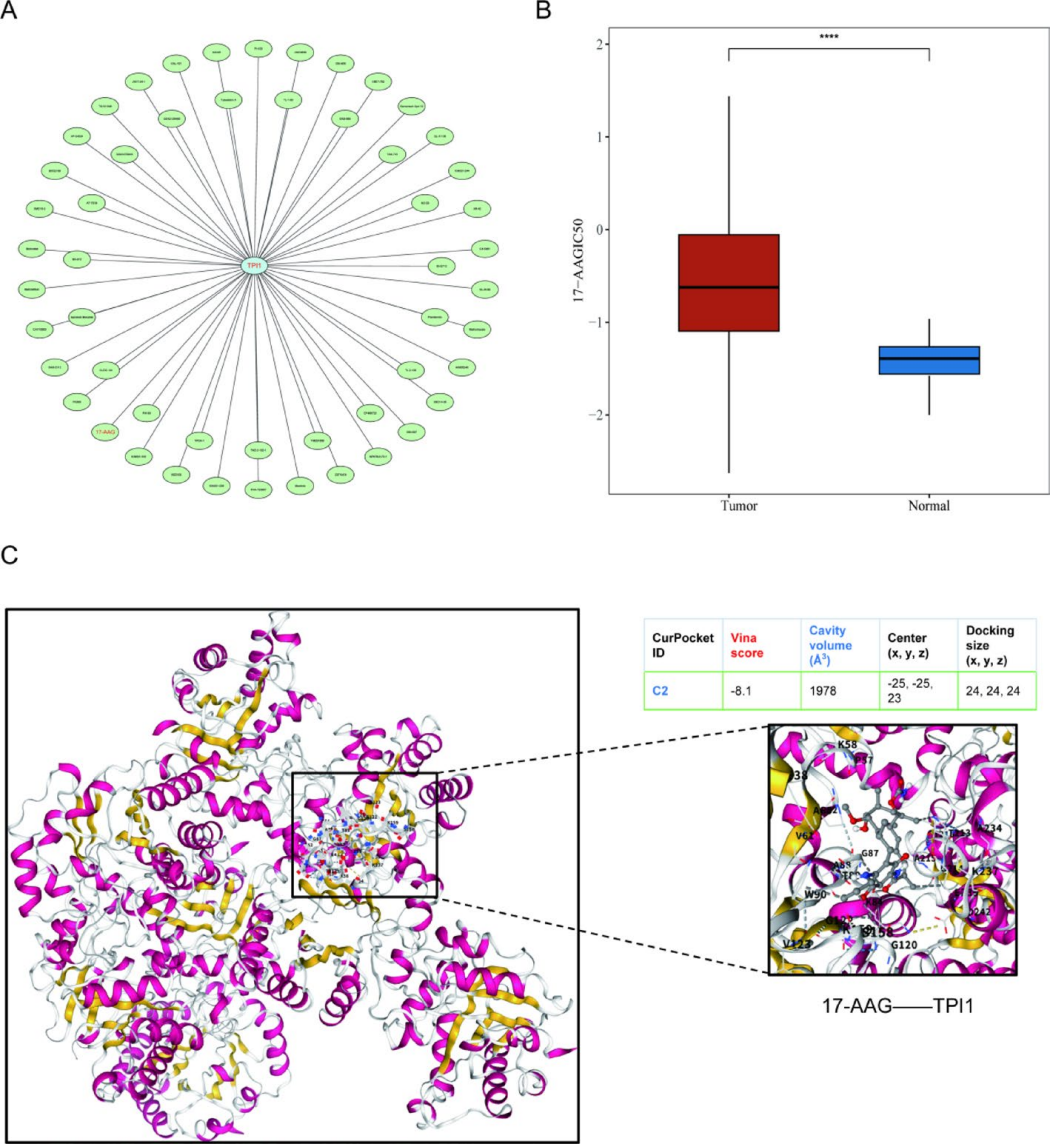
Drug prediction for the treatment of LUAD patients targeting TPI1. **A** Predicted drug–target interaction network of TPI1. **B** Drug sensitivity analysis of TPI1 in LUAD. **C** Molecular docking validation of TPI1 and 17-AGG

To further investigate the therapeutic potential of TPI1 as a druggable target, we performed systematic drug sensitivity analysis in LUAD patients. The results revealed that the HSP90 inhibitor 17-AAG exhibited significantly higher sensitivity in LUAD tissues with elevated TPI1 expression, suggesting it as a promising candidate therapeutic agent targeting TPI1 (Fig. 7A, B). This finding was subsequently validated by molecular docking analysis, which demonstrated that 17-AAG bound to TPI1 with high affinity (binding energy = − 8.1 kcal/mol) (Fig. 7C). Collectively, these results indicate that 17-AAG may exert anti-tumor effects by directly targeting TPI1, thereby providing a novel candidate drug and theoretical basis for LUAD targeted therapy.

### Novel CM-related consensus subgroups identification in LUAD patients

Based on 8 CM -associated DEGs, we performed consensus clustering analysis on the TCGA-LUAD cohort, which classified patients into two distinct subgroups (C1 and C2) (Fig. 7A, B and D). Further differential analysis revealed significant differences in DEGs between the 2 subtypes (Fig. 7C). Moreover, KM survival analysis demonstrated that patients in the C1 subgroup had significantly better overall survival compared to those in the C2 subgroup (Fig. 7E), indicating the prognostic relevance of this molecular classification.

**Fig. 8:**
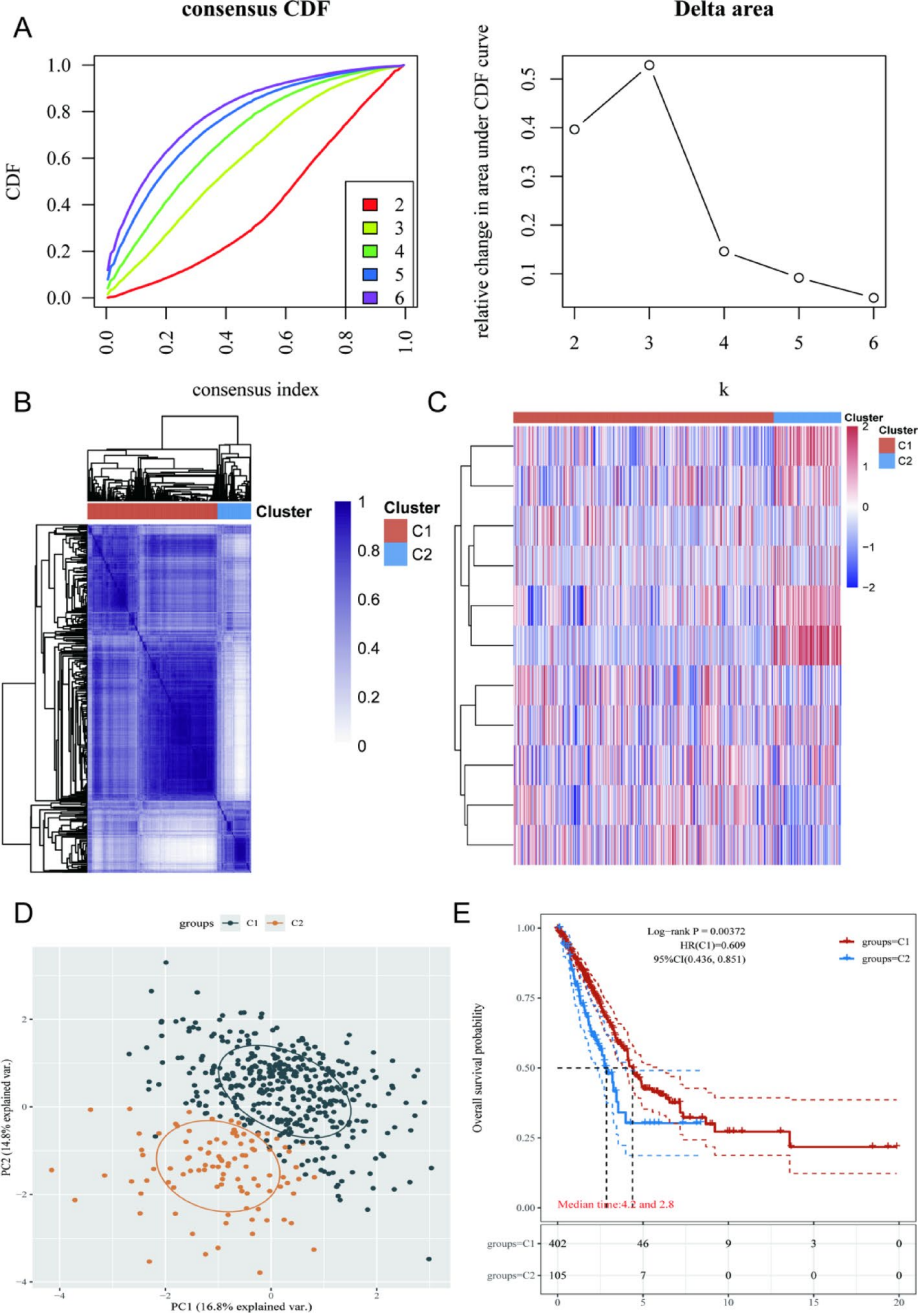
COPD-MEM associated molecular subgroups identification in LUAD patients. **A** Consensus CDF plot of consensus clustering result. **B** Consensus clustering identified 2 stable molecular subtypes (C1 and C2) in LUAD based on CM-related genes. **C** Heatmap illustration of DEGs in C1 and C2 subgroups. **D** PCA plot of C1 and C2. **E** KM plot of C1 and C2

### Difference between CM-related molecular subgroups

**Fig. 9:**
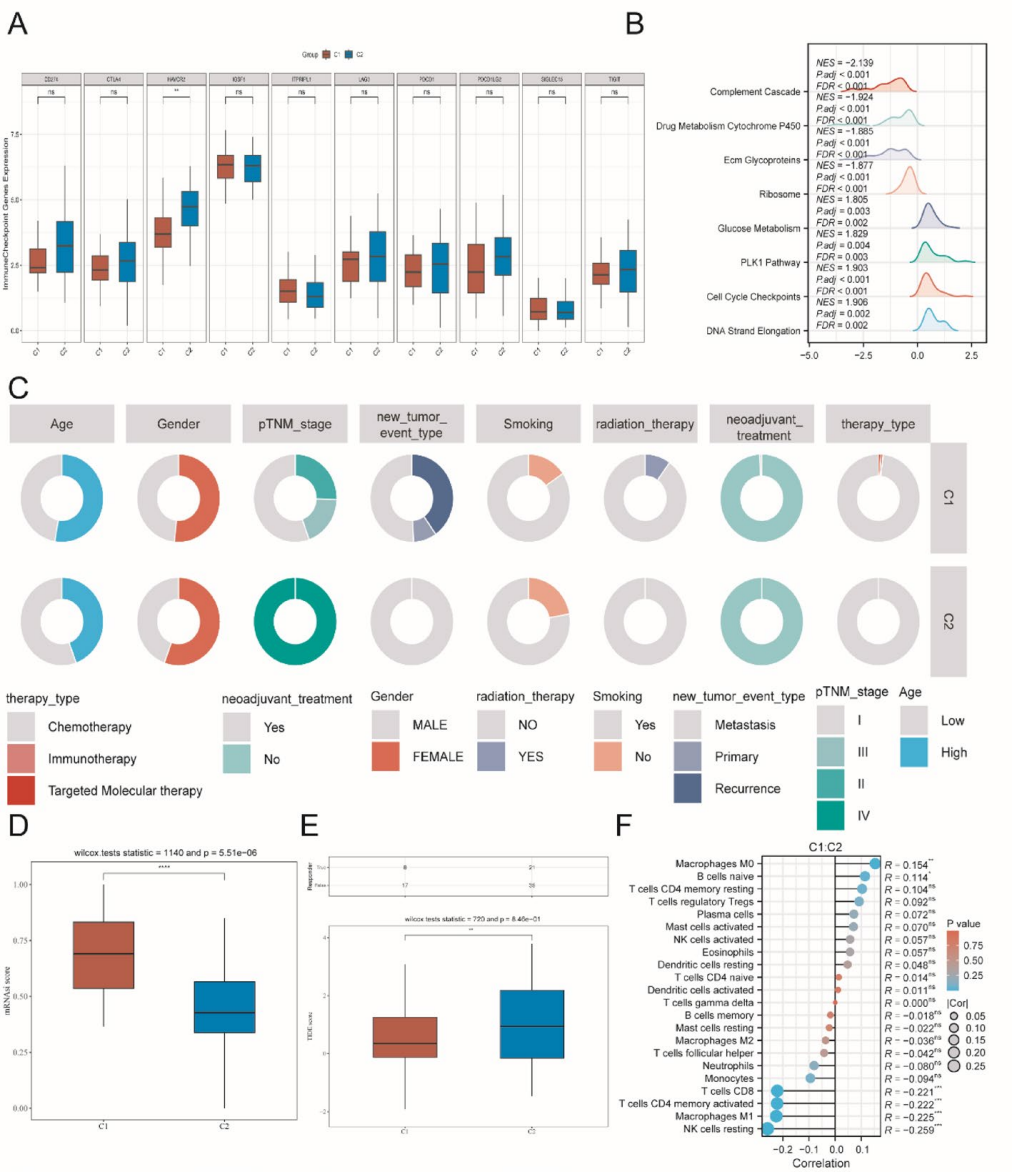
Molecular and immune differences between different CM-associated molecular subgroups. **A** Immune checkpoint difference between C1 and C2. **B** Molecular and biological function differences between C1 and C2. **C** Clinical parameter difference between C1 and C2. **D** Tumor stemness comparison between C1 and C2. **E** TIDE enrichment score between C1 and C2. **D** Tumor stemness comparison between C1 and C2. **F** Immune infiltration analysis between C1 and C2

We first compared immune checkpoint gene expression and immune infiltration between the C1 and C2 subgroups. The results revealed marked differences, particularly in HAVCR2 expression, the abundance of multiple immune cell types, and the activation of immune response pathways (Fig. 8A and F). In addition, the C1 subtype exhibited higher tumor stemness (elevated mRNAsi), whereas the C2 subtype displayed stronger immune evasion characteristics (higher TIDE score) (Fig. 8D-E). Besides, we also compared the clinical difference and molecular function difference between C1 and C2 (Fig. 8B-C). These findings suggest that the 2 subtypes may differ in molecular and heterogeneity (Fig. 9).

### The association of TPI1 with LUAD tumor progression

**Fig. 10:**
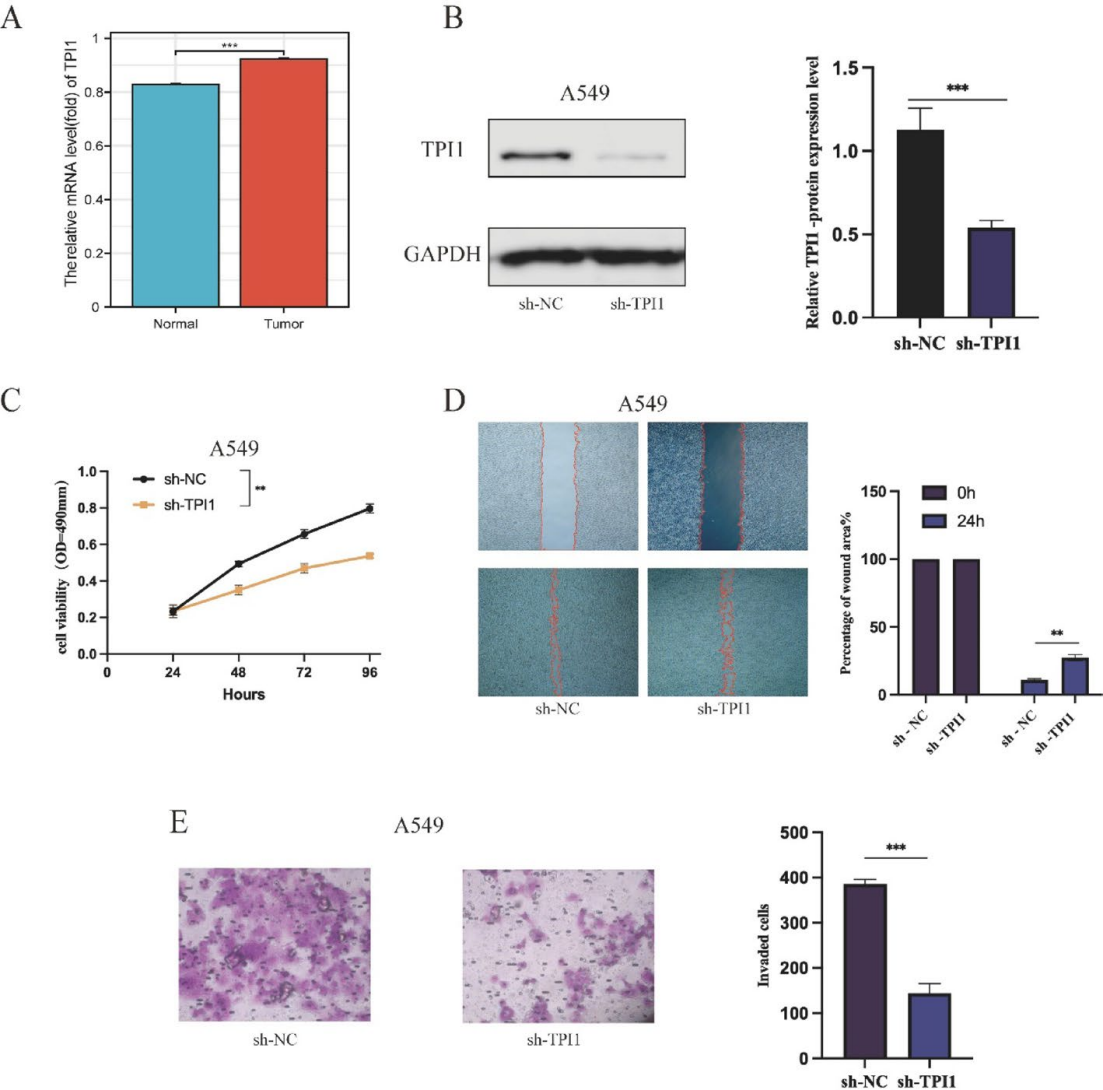
Functional validation of TPI1 knockdown in A549. **A** The mRNA expression level examination of LUAD cancer cell lines compared to normal cell lines. **B** WB analysis confirmed efficient knockdown of TPI1 by silencing vector in A549 cells. **C** Cell proliferation assays following A549 knockdown in A549 cells. **D** Wound-healing assays following TPI1 knockdown in A549 cells. **E** Transwell invasion assays following TPI1 knockdown in A549 cells. Data are presented as mean ± SD, *p < 0.05, **p < 0.01, ***p < 0.001

To further elucidate the functional role of TPI1 in LUAD, we performed a series of in vitro experiments employing the A549 and BEAS-2B cell lines. Notably, the mRNA expression level of TPI1 was found to be elevated in A549 cells relative to BEAS-2B cells (Fig. 10A). Additionally, Western blot analysis demonstrated that the expression of TPI1 was significantly reduced through the use of shRNA (Fig. 10B). CCK-8 assays indicated that the downregulation of TPI1 markedly diminished the proliferative capacity of A549 cells when compared to the sh-NC control group (Fig. 10C). Similarly, wound-healing assays revealed a notable reduction in the migratory abilities of the cells following TPI1 knockdown (Fig. 10D). Furthermore, Transwell invasion assays showed a significant decrease in the invasive potential of cells deficient in TPI1 in comparison to the control group (Fig. 10E). Collectively, these findings highlight the contribution of TPI1 in promoting the proliferation, migration, and invasion of LUAD cells, thereby emphasizing its potential as an oncogenic driver.

## Discussion and conclusion

With the advancement of integrative bioinformatic pipelines, investigations have been successfully constructed predictive models and deciphered the tumor heterogeneity for LUAD patients. For examples, integrating machine learning and multi-omics analysis identified Treg-associated programmed cell death(PCD) features and crotonylation-associated prognostic model for clear cell renal cell carcinoma(CCRC) patients [45, 46]. Besides, other studies also successfully identified the molecular relationship between atherosclerosis and LUAD [47]. Similarly, an independent investigation also pointed out the mitotic catastrophe heterogeneity and absorption, distribution, metabolism, excretion (ADME)-associated predictive model for bladder cancer patients [48, 49]. However, there still has not yet been reported the mechanisms and predictive model targeting CM for LUAD patients. In this study, we developed a comprehensive CM-related predictive and therapeutic frameworks for forecasting clinical outcomes of LUAD patients via integrative bioinformatic approaches and multi-omics. Besides, we also highlighted the TPI1 character in LUAD pathogenesis by in silico and in vitro assays.

Mechanistically, our findings highlighted the tight pathological connection among LUAD with CM. COPD, an independent risk factor for LUAD, drives chronic inflammation, oxidative stress, and airway remodeling that not only sustain lung tissue injury but also create a permissive environment for genetic and epigenetic alterations in tumor cells [50]. Lung epithelial and immune cells in COPD patients frequently exhibit mitochondrial dysfunction, including impaired OXPHOS, excessive mitochondrial reactive oxygen species (mtROS) production, and defective mitophagy, which together promote persistent inflammation, DNA damage, and malignant transformation, ensuing leading to the pathogenesis of LUAD [51]. At the same time, TPI1, a key enzyme in the glycolytic pathway that catalyzes the interconversion of dihydroxyacetone phosphate and glyceraldehyde-3-phosphate, playing an essential role in maintaining cellular energy supply and metabolic balance, was pointed out as CM-associated hub gene involved in LUAD pathogenesis. Previous reports indicated the tumor-promoting role of TPI1 in LUAD [52–54]. Besides, in our study, we highlighted the CM role of TPI1 in LUAD pathogenesis, which provides additional idea in LUAD pathophysiology.

In conclusion, our study first pointed out CM-associated predictive model for LUAD patients and pointed out TPI1 pathogenic role of TPI1 and therapeutic potential of 17-AAG in LUAD. However, there are still limitations in our study. For examples, the model performance of our study should be validated in the larger clinical cohort. Besides, the detailed molecular mechanisms of TPI1 in regulation of CM and LUAD pathogenesis should be elucidated in pre-clinical studies. In addition, the therapeutic efficacy and safety of 17-AAG should be validated in pre-clinical and clinical trials.

## Data Availability

The datasets used and analyzed during the current study are publicly available from the Gene Expression Omnibus (GEO) and The Cancer Genome Atlas (TCGA) databases. Specific access details for each dataset are provided as follows: GSE32863: Accession number GSE32863, direct URL: https://www.ncbi.nlm.nih.gov/geo/query/acc.cgi? acc=GSE32863TCGA-LUAD: Project accession TCGA-LUAD, direct URL: https://portal.gdc.cancer.gov/projects/TCGA-LUADGSE13213: Accession number GSE13213, direct URL: https://www.ncbi.nlm.nih.gov/geo/query/acc.cgi? acc=GSE13213.

## Code availability

The underlying code for this study is not publicly available but may be made available upon reasonable request from the corresponding author.
